# Jinggangmycin-Induced UDP-Glycosyltransferase 1-2-Like Is a Positive Modulator of Fecundity and Population Growth in *Nilaparvata lugens* (Stål) (Hemiptera: Delphacidae)

**DOI:** 10.3389/fphys.2019.00747

**Published:** 2019-06-21

**Authors:** Lin Quan Ge, Sui Zheng, Hao Tian Gu, Yong Kai Zhou, Ze Zhou, Qi Sheng Song, David Stanley

**Affiliations:** ^1^School of Horticulture and Plant Protection, Yangzhou University, Yangzhou, China; ^2^Division of Plant Sciences, University of Missouri, Columbia, MO, United States; ^3^Biological Control of Insects Research Laboratory, United States Department of Agriculture – Agricultural Research Service, Columbia, MO, United States

**Keywords:** *Nilaparvata lugens*, UDP-glycosyltrasferase 1-2-like, jinggangmycin, fecundity, population growth

## Abstract

The antibiotic jinggangmycin (JGM) is broadly applied in Chinese rice producing regions to control rice blight, a fungal disease. Aside from protecting rice plants from the disease, JGM leads to the unexpected action of stimulating brown planthopper (BPH; *Nilaparvata lugens*; Hemiptera: Delphacidae) reproduction to the extent it can influence population sizes. The JGM-induced BPH population growth has potential for severe agricultural problems and we are working to understand and mitigate the mechanisms of the enhanced reproduction. UDP-glucuronosyltransferases (UGTs) are multifunctional detoxification enzymes responsible for biotransformation of diverse lipophilic compounds. The biological significance of this enzyme family in insect fecundity is not fully understood, however, upregulated *UGT12* in JGM-treated BPH, may influence fecundity through metabolism of developmental hormones. This idea prompted our hypothesis that *NlUGT12* is a positive modulator of BPH reproductive biology. JGM treatment led to significant increases in accumulations of mRNA encoding *Nl*UGT12, numbers of eggs laid, oviposition period, juvenile hormone III titers, and fat body, and ovarian protein contents. dsUGT12 treatment suppressed *NlUGT12* expression and reversed JGM-enhanced effects, resulting in under-developed ovaries and reduced expression of juvenile hormone acid methyltransferase and the JH receptor, methoprene tolerant. Application of the JH analog, methoprene, on dsUGT12 treated-females partially reversed the dsUTG12 influence on vitellogenin synthesis and on *NlUGT12* expression. These results represent an important support for our hypothesis.

## Introduction

The brown planthopper (BPH), *Nilaparvata lugens* (Stål) (Hemiptera: Delphacidae), is a classic pesticide-induced resurgence insect pest. The fungicide jinggangmycin (JGM), a product of *Streptomyces var. jinggangen*, is applied for rice sheath blight (*Rhizoctonia solani*) control in China (Peng et al., 2014). JGM foliar sprays alter BPH male and female physiological parameters and enhance fecundity, by up to 99%. A few examples of these physiological changes include enhanced flight capacity, thermotolerance, body weights, protein and lipid contents, and JH titers (Jiang et al., 2012). It is an attractive product due to its low cost, efficacy in the field, low toxicity and environmental residue. The current concern is that it also has real potential to spark agroecological catastrophes that may follow JGM-stimulated BPH fecundity.

JGM increased BPH fecundity via increased fatty acid metabolism (Jiang et al., 2016; Li et al., 2016), however, the broad impact of JGM led us to consider additional pathways that might also influence BPH reproduction. From digital gene (DEG) expression profiles, we identified a UDP-glycosyltransferase 1-2 like (*UGT12*) gene that was up-regulated in adult females following foliar JGM treatments (200 ppm) at 2 days after emergence (2 DAE). The UGTs form a superfamily of enzymes that catalyze the transfer of a glycosyl group from a nucleotide sugar, such as UDP-glucuronic acid or UDP-glucose, to a variety of small hydrophobic molecules (aglycones), thereby making them hydrophilic for efficient excretion (Mackenzie et al., 1997; Meech and Machenzie, 1997; Meech et al., 2019). Members of this superfamily are ubiquitous and act in many biological processes, including olfaction (Lazard et al., 1991), steroid metabolism, and detoxification of exogenous substrates (Meech and Machenzie, 1997).

In insects, these enzymes typically utilize UDP-glucose as the sugar donor (Ahmad and Hopkins, 1993a). As in vertebrates, endogenous and exogenous substrates undergo glycosylation in insects. Insect UGTs act in detoxification of plant allelochemicals, cuticle formation, pigmentation, olfaction, and ecdysteroid (20E) metabolism (Tompson et al., 1987; Hopkins and Kramer, 1992; Ahmad and Hopkins, 1993a,b; Wang et al., 1999). The UGT enzyme activity has been reported in tissues, such as fat body, of many insect orders, including fruit flies, *Drosophila melanogaster* (Diptera: Drosophilidae) (Real et al., 1991). The presence of UGT enzymes in the antenna of *D. melanogaster* indicate a role in olfaction (Wang et al., 1999). UGT activity was also recorded in silkworms, *Bombyx mori* (Lepidoptera: Bombycidae) (Luque et al., 2002). Many endogenous compounds, like 20E (Svoboda and Weirich, 1995) and cuticle tanning precursors (Hopkins and Kramer, 1992; Ahmad et al., 1996) are glycosylated by UGTs. UGTs in *Helicoverpa armigera* Hübner (Lepidoptera: Noctuidae) are factors for host plant adaptation and insecticide resistance (Heckel, 2010). Li et al. (2016) found that *UGT2B17* operates in chlorantraniliprole resistance in *Plutella xylostella* (L.) (Lepidoptera: Plutellidae).

It remains unknown whether UGT-encoding genes act in insect reproduction, although they may act indirectly. Collier et al. (2012), for example, reported that UGT1a genes are expressed in murine placentas and fetal livers in a model of intracytoplasmic sperm injection and *in vitro* fertilization. These genes promote enhanced steroid hormone clearance, which may mediate negative outcomes. In their review of the UGT superfamily, Meech et al. (2019) note that UGT-encoding genes are expressed in a range of reproduction-functioning tissues, including ovary, uterus, cervix, placenta, breast, testes, and prostate. We infer UGTs influence reproductive events by catalyzing various metabolic steps that influence levels of steroid hormones and other biomolecules. From this, we posed the hypothesis that NlUGT12 acts as a positive modulator of BPH reproductive biology. In this paper we report on the outcomes of experiments designed to test our hypothesis.

## Materials and Methods

### Rice Variety, Insect, and Pesticide

Rice seeds (*Oryza sativa* L.) of the variety Ningjing 2 (Japonica rice) were sown in rectangular field cement tanks (200 cm × 100 cm × 60 cm, L, W, H). Rice plants at the tillering stage were used for all experiments.

The *Nilaparvata lugens* strain was obtained from the China National Rice Research Institute (CNRRI: Hangzhou, China). BPH were reared on rice plants in cement tanks covered with fine mesh under natural conditions from April to October and thereafter overwintered in an insectary at 26 ± 2°C and 14L:10D at Yangzhou University. Technical grade JGM (C_20_H_35_O_13_N) (61.7% a.i) (Qianjing Biochemistry Co. Ltd., Haining, Zhejiang Province, China) was used in all trials. Third instar BPH was used in all experiments (Ge et al., 2009; Wang et al., 2010).

### Experimental Design

Rice plants were sprayed with 200 ppm JGM and control plants were treated with tap water. Each treatment was repeated three times (3 pots) and distributed randomly. The 72 h-post-spray nymphs were selected for RNAi experiments and maintained in a climate chamber (Model: RXZ 328A) (Jiangnan Instrument Factory, Ningbo, Zhejiang, China) at 26 ± 2°C and L16:D8 in glass jars (6-cm diameter, 12-cm height) with a rice stem until emergence.

### dsRNA Synthesis

Based on our digital gene (DEG) expression profiles, we selected UGT12 (accession no. XM022331295) as a RNAi target in the JGM-treated BPH. To synthesize double-stranded RNA (dsRNA), a 575 bp (the fragment from 861 to 1435 bp) was amplified with T_7_ RNA polymerase promoter linked primers (Table 1). The thermocycler (T-100, Bio-Rad Co., California, United States) was programmed at pre-denaturing at 94°C for 4 min, followed by 35 cycles of denaturing at 94°C for 30 s, annealing at 55.2°C for 30 s and extension at 72°C for 1 min, with a final extension at 72°C for 10 min. The products were sequenced and then used as queries in BLAST searches to control identity in NCBI (98–99% identity) prior to dsRNA synthesis. The GFP gene (accession no. ACY56286) was used as a negative control (Chen et al., 2010). All dsRNAs were synthesized using a T_7_ RiboMAX^TM^ Express RNAi System (Promega, Madison, WI, United States) according to the manufacturer’s protocols. The dsRNA products were diluted with 50 μL diethylpyrocarbonate-treated water and stored at -80°C. The purified dsRNAs were quantified using spectroscopy at 260 nm and separated by agarose gels to validate their integrity.

**Table 1:** PCR primers used in this study.

Purpose	Primer ID	Sequence (5′-3′)	Size (bp)
qPCR analysis	Q-UGT12-F	ACACTGCCTACGAACTGGAA	157
	Q-UGT12-R	CTCCATCCTCTGACTCGTCC	
	Q-Vg-F	GCTTGTCAGAATGCCACC	184
	Q-Vg-R	TCTTGCCAGAAGGATTGC	
	Q-VgR-F	AGGCAGCCACACAGATAACCGC	136
	Q-VgR-R	AGCCGCTCGCTCCAGAACATT	
	Q-JHAMT-F	AAATACGGCAATAAGAAC	112
	Q-JHAMT-R	GAAGACAATAAAACGAGA	
	Q-Met-F	CCGCACCCAACAACAATACA	288
	Q-Met-R	CCAATCCGTTTACCACCACA	
	actin-F	TGCGTGACATCAAGGAGAAGC	186
	actin-R	CCATACCCAAGAAGGAAGGCT	
dsRNA	UGT12-F	GGACGAGTCAGAGGATGGAG	575
synthesis	UGT12-R	AGGTGAGATCAAAGCAGGCT	
	UGT12-T7F	Taatacgactcactataggg (T_7_ promoter)	
		GGACGAGTCAGAGGATGGAG	
	UGT12-T7R	Taatacgactcactataggg (T_7_ promoter)	
		AGGTGAGATCAAAGCAGGCT	
	GFP-F	AAGGGCGAGGAGCTGTTCACCG	688
	GFP-R	CAGCAGGACCATGTGATCGCGC	
	GFP-T7F	Taatacgactcactataggg (T_7_ promoter)	
		AAGGGCGAGGAGCTGTTCACCG	
	GFP-T7R	Taatacgactcactataggg (T_7_ protometer)	
		CAGCAGGACCATGTGATCGCGC	


F, forward; R, reverse. Lowercase letters labeled sequences represent for T_7_ promoter.

### Silencing UGT12

BPH were released into potted rice and treated with JGM as just described. The dsRNA treatments induced high mortality of second instar nymphs (over 95%) (Ge et al., 2017). Third-instar nymphs (20/treatment) were collected 3 days after the JGM treatments, and subjected to RNAi via an artificial diet as described by Dong et al. (2011), with slight modifications. Earlier studies found a high efficiency of dsRNA ingestion-mediated gene silencing in BPH (Fu et al., 2001). Glass cylinders (15.0-cm L × 2.5-cm D) have been used as feeding chambers, with four dsRNA concentrations, 0.12, 0.075, 0.05, and 0.025 μg/μL. In this study, the optimal dsRNA concentration, 0.075 μg/μL, was determined from preliminary experiments with the noted concentrations. The artificial diet (40 μL) was held between two layers of stretched Parafilm M membrane enclosing the two open ends of the chamber (the diet capsule), which was replaced every second day. Six days later, fifth (final) instar nymphs were transferred into a glass jar (12-cm H × 6-cm D), and maintained on rice plants under 26 ± 2°C, RH 90%, and 16L:8D photoperiod. The newly emerged females were collected separately and soluble protein content (fat body and ovary, 15 BPH/replicate or *n* = 15, *N* = 3 replicates), JH (whole body, *n* = 5, *N* = 3) and ecdysone titers (whole body, *n* = 5, *N* = 3), body weight (*n* = 10, *N* = 3), and longevity (*N* = 19) were recorded. We used whole bodies to determine the expression levels of *NlVg*, *NlVgR* (vitellogenin receptor, VgR), *NlJHAMT*, *NlMet*, and *NlUGT12* (*n* = 5, *N* = 3 for each gene) at 2 or 3 DAE and similarly whole bodies were used to determine Vg, JHAMT, Met, and UGT12. All the replicates were biologically independent. We separately selected 3 groups, 10 virgin females/group, to determine *NlUGT12* RNAi efficiency at 1, 3, 5, and 7 DAE. Others were used to isolate reproductive tracts at 2, 4, and 6 DAE. We recorded the pre-oviposition period, oviposition period, adult female longevity, and fecundity of 20 mated pairs (untreated ♀ × ♂). The eggs laid were counted under a microscope and rice stalks were replaced every second day.

### Extraction and cDNA Synthesis

Total RNA was extracted using the Trizol reagent (Invitrogen, Carlsbad, CA, United States) according to the manufacturer’s instructions. The concentration and purity of RNA sample were measured using a Nanodrop 2000C spectrophotometer (Thermo Fisher Scientific, West Palm Beach, FL, United States) and the integrity was checked by 1.5% agarose gel electrophoresis. Genomic DNA was cleared bussing DNase treatment. The first-strand cDNA was synthesized from 1 μg of the total RNA using a PrimeScript RT Reagent Kit with gDNA Eraser (TaKaRa, Tokyo, Japan) in a final volume of 20 μL.

### Quantitative Real-Time PCR (qPCR)

Primers for qPCR were designed at http://primer3.ut.ee/ (Rozen and Skaletsky, 2000) (Table 1). Primer sets were validated by relative standard curves for each gene transcript and amplification efficiency (*E*) along with correlation coefficient (*R^2^*) were calculated for each primer pair. All qPCR reactions were performed in biologically independent triplicates in a CFX96 real-time PCR system (Bio-Rad Co., Ltd., California, United States). Each 10 μL reaction contained 5 μL 2 × SYBR Premix^EX^ TaqII Master Mix (TaKaRa, Tokyo, Japan), 0.4 μL reverse primer (10 μM), 1 μL cDNA template and 3.2 μL of deionized water. The thermocycler was programmed at 95°C for 30 s, followed by 35 cycles at 95°C for 5 s, 55.2°C for 15 s, and 72°C for 1 min. A β-actin (EU179846) cDNA fragment was amplified with actin-F and actin-R primers (Table 1) as a reference gene (Chen et al., 2010). The relative mRNA levels of target genes were quantified using the 2^-ΔΔCt^ method (Livak and Schmittgen, 2001). Three biologically independent replicates based on independent RNA sample preparations were performed for each gene.

### Effects of Dietary dsRNA on Biological Performance Parameters

Protein contents in the fat bodies and ovaries were determined using the Bradford protocol (Bradford, 1976; Gong et al., 1980) with Coomassie Brilliant Blue G 250 (Shanghai Chemical Agent Co., Ltd., Shanghai, China). The fat bodies and ovaries were isolated under a zoom-stereomicroscope (model XTL20, Beijing Tech Instrument Co., Ltd., Beijing, China) in a cooled petri dish. The isolated ovaries and fat bodies were placed in pre-weighed separate ice-cold centrifuge tubes. The tubes were re-weighted on a Mettler-Toledo electronic balance (EC 100 model; 1/10000 g sensitivity), homogenized on ice in 5 mL phosphate buffered saline (PBS; 137 nM NaCl; 2.68 nM KCl; 1.47 mM KH_2_PO_4_; and 8.10 mM Na_2_HPO_4_, pH 7.0), and centrifuged at 10,000 × g at 4°C for 20 min. The supernatants were collected as sample preparations. A standard curve was established based on a standard protein (bovine serum albumin (Shanghai Biochemistry Research Institute, Shanghai, China). The absorbance at 595 nm was determined in a UV755B spectrometer (Shanghai Precision Instrument Co., Ltd., Shanghai, China). The protein content in the sample solution was calculated from the standard curve.

For body weight measurement, 10 females at 2 DAE were used as a replicate for each treatment, and control group. Females were placed in pre-weighed centrifuge tubes and weighed on the balance just described. The treatment and control groups had three biologically independent replicates.

We followed the manufacturer’s instructions to determine JH III (*n* = 5, *N* = 3) and ecdysone titers (*n* = 5, *N* = 3) of whole body using an insect double sandwich ELISA kit (Qiaodu biological technology Co., Ltd., Shanghai, China). Each treatment was performed in biologically independent triplicates.

### Hormone Treatment

Methoprene (Sigma Chemical Co., Ltd.,cn, United States, weight, 100 mg, Purity, 99.5%) was dissolved in 1 mL acetone to produce a 100 μg/μL stock solution, and stored at -20°C (Ayoade et al., 1999). The stock solutions were diluted to concentrations indicated in Results with acetone and topically applied to the backs of newly emerged, RNAi-treated (100 ng/μL) females using a syringe (Terumo), and a micro applicator (Burkard). The BPH were transferred into glass jars described above and maintained on rice plants at 26 ± 2°C, RH 90%, and 16L:8D photoperiod. The insects were collected at 24 and 48 h, and *NlVg* and *NlUGT12* expression levels, and Vg protein synthesis post methoprene administration were determined. Each treatment was performed in biologically independent triplicates.

### Ovary Isolation

The ovaries were isolated from the 2, 4, and 6 DAE females. There were four treatment groups: untreated controls, JGM-treated, JGM+dsGFP-treated, and JGM+dsUGT12-treated in 1 × PBS, *n* = at least 10 ovaries/group. After extensive washing, ovaries were photographed with a Leica DMR connected to a Fuji FinePix S_2_ Pro digital camera (Germany).

### Western Blot Analysis

Isolated fat bodies were homogenized in precooled lysis buffer (8 M urea, 4% CHAPS, 40 nM Tris pH 8.0, 5 nM EDTA, 1 mM PMSF, and 10 mM DTT) (Amersco, Solon, OH, United States) and 0.2 mM protease inhibitor (Roche Diagnostics, Basel, Switzerland), held at 4°C for 1 h and centrifuged at 12,000 ×*g* at 4°C for 20 min. Protein concentration was detected using a bicinchoninic acid (BCA) method (CWOO14S, CWBIO Biological Co., Taizhou, Jiangsu Province, China). Aliquots of 30 μg protein were separated by 8% SDS-PAGE, electroblotted (Model: 1658001, Bio-Rad, Co., Ltd., California, United States) onto polyvinglidene fluoride membrane (PVDF) (Millipore, Billerica, MA, United States). After blocking with 5% non-fat milk (Amersco, Solon, OH, United States) in Tris–buffered saline (TBS) for 2 h at room temperature, the membranes were incubated with *N. lugens* primary antibodies for *β-actin* (1:2000) and *NlVg* (1:5000) at 4°C overnight. After washing three times for 15 min each with 0.5% Tween-20 in TBS (TBST), the membranes were incubated with goat anti-rabbit immunoglobulin G horseradish peroxidase-conjugated secondary antibody (Sigma, St. Louis, MO, United States) for 1 h at room temperature (Lu et al., 2016). The immunoreactivity was detected using the SuperSignal West Pico Chemiluminescent Substrate ECL (Pierce, Rockford, IL, United States) and the membrane was imaged with a GBOX-Chemi XT4 Imager (Syngene, Cambridge, United Kingdom). Vg protein abundance was compared with the internal control, β-actin. The Vg antibody was a gift from Professor Qiang Zhou (Sun Yat-sen University). The mean gray values of each band were normalized to β-actin using NIH image J software^1^, *n* = 3 biologically independent replicates (Supplementary Material).

### Population Growth

We set up four mating groups: 1/ controls, untreated males × untreated females; 2/ control-♂ × JGM-treated females (JGM-♀); 3/ control-♂ × JGM+dsGFP-treated females (JGM+dsGFP-♀); and 4/ control-♂ × JGM+dsUGT12-treated females (JGM+dsUGT12-♀). The experiment was arranged with a randomized complete block design with five biologically independent replicates. Two pairs of newly emerged BPH were released onto rice plants and covered with an 80-mesh nylon cylindrical cage (20 cm D × 80 cm H) in each pot. Ten days later, we started counting the next generation neonates each day, which were transferred into new plastic plots with rice plants covered as described until the parent females died. Numbers of neonates in the new plastic plot were recorded every second day until adult emergence. Numbers of unhatched eggs on the rice stems were recorded. Hatching rate was also recoded (total neonates/total neonates plus unhatched eggs). The population growth index (PGI) was expressed as N1/N0, with N1 representing the total neonates of next generation and unhatched eggs, and N0 representing the insects released (N0 = 4).

### Statistical Analysis

**FIGURE 1:**
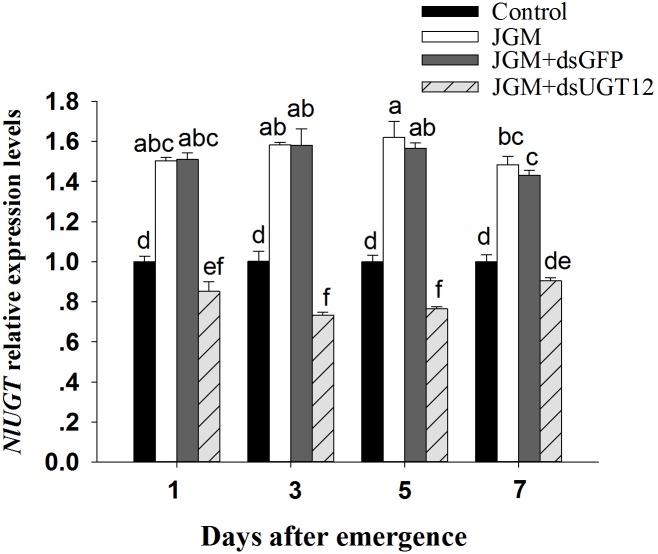
Effects of JGM+dsUGT12 treatments on expression levels at different days post emergence (DAE). *NlUGT12* expression value of untreated females was converted to 1. The histogram bars show mean relative gene expression (*n* = 3 independent biological replicates) and the error bars represent one standard deviation (Tukey, *P* < 0.05). Gene expression was normalized to the β-actin reference gene.

The normal distributions and homogeneity of variances (determined using the Bartlett test) were verified before performing analysis of variance (ANOVA) tests. A one-way ANOVA was performed for the number of eggs laid (*N* = 19), the pre-oviposition period (*N* = 19), the oviposition period (*N* = 19), the longevity of the females (*N* = 19), the contents of soluble protein (fat body and ovary, *n* = 15, *N* = 3), JH III and ecdysone titer (*n* = 5, *N* = 3), body weight (*n* = 10, *N* = 3), longevity (*N* = 19), the number of offspring (*N* = 5), hatching rate (*N* = 5), gender ratio (*N* = 5), western blot (*n* = 20, *N* = 3), and *NlVg, NlVgR, NlJHAMT, NlMet* and *NlUGT12* expression levels (*n* = 5, *N* = 3). All data were expressed as means ± standard error of the mean (SEM) from three independent biological replicates, unless otherwise noted in figure legends. Two-way (days after emergence and dsRNA treatment) ANOVAs were performed to analyze the data in Figure 1. Multiple comparisons of the means were conducted using Fisher’s Protected Significant Difference (PLSD) test. All analyses were conducted using the data processing system (DPS) of Tang and Feng (2002). Biological parameter data were analyzed using an analysis of variance (ANOVA) with one factor and statistical data are presented in Table 2.

**Table 2:** Statistical analyses of biological parameter data.

Experiment	Statistic
dsUGT12 reduced *NlUGT12* expression at different days after emergence	*F* = 1030.8, df = 3, 47, *P* = 0.0001
Effects of dsUGT12 silencing on *NlUGT12* expression at DAEs	*F* = 1.4, df = 3, 47, *P* = 0.2552
Effects of dsUGT12 silencing on interactive effect between DAEs and JGM+dsRNA treatments	*F* = 8.8, df = 3, 47, *P* = 0.0001
dsUGT12 reduced JH titer, 2 DAE	*F* = 34.2, df = 3, 11, *P* = 0.0001
dsUGT12 increased 20E titer, 2 DAE	*F* = 130.6, df = 3, 11, *P* = 0.0001
dsUGT12 reduced protein content of fat body and ovary, 2 DAE	*F* = 36.9, df = 3, 11, *P* = 0.0001 for fat body *F* = 21.4, df = 3, 11, *P* = 0.0004 for ovary
dsUGT12 reduced *JHAMT* and *Met* expression, 2 DAE	*F* = 325.3, df = 3.11, *P* = 0.0001 for *JHAMT* *F* = 75.4, df = 3, 11, *P* = 0.0001 for *Met*
dsUGT12 reduced body weight, 2 DAE	*F* = 30.1, df = 3, 11, *P* = 0.0001
dsUGT12 reduced number of laid eggs	*F* = 39.3, df = 3, 75, *P* = 0.0001
dsUGT12 reduced oviposition period	*F* = 23.5, df = 3, 75, *P* = 0.0001
dsUGT12 was not significant difference for preoviposition period	*F* = 2.1, df = 3, 75, *P* = 0.1077
dsUGT12 was not significant difference for longevity of females	*F* = 0.659, df = 3, 75, *P* = 0.5802
dsUGT12 reduced *NlVg* expression, 2DAE	*F* = 74.6, df = 3, 11, *P* = 0.0001
dsUGT12 reduced *NlVg* expression, 3 DAE	*F* = 23.5, df = 3, 11, *P* = 0.0003
dsUGT12 reduced *NlVgR* expression, 2 DAE	*F* = 16.3, df = 3, 11, *P* = 0.0009
dsUGT12 reduced *NlVgR* expression, 3 DAE	*F* = 27.9, df = 3, 11, *P* = 0.0001
dsUGT12+methoprene rescued *NlUGT12* expression, 24 h	*F* = 59.2, df = 4, 14, *P* = 0.0001
dsUGT12+methoprene rescued *NlUGT12* expression, 48 h	*F* = 99.8, df = 4, 14, *P* = 0.0001
dsUGT12+methoprene rescued *NlVg* expression, 24 h	*F* = 39.6, df = 4,14, *P* = 0.0001
dsUGT12+methoprene rescued *NlVg* expression, 48 h	F = 66.5, df = 4, 14, *P* = 0.0001
dsUGT12 reduced number of offspring	*F* = 16.9, df = 3, 19, *P* = 0.0001
dsUGT12 reduced eggs hatching rate	*F* = 18.6, df = 3, 19, *P* = 0.0001
dsUGT12 let to no significant difference for gender ratio	*F* = 1.62, df = 3, 19, *P* = 0.1847

## Results

### dsUGT12 Treatments Reduce JGM-Enhanced NlUGT12 Expression

Compared to untreated controls, the JGM treatments led to substantially increased accumulations of mRNA encoding NlUGT12 over the 7-day experiment. The dsUGT12 treatments reversed the enhancing influence of JGM, with accumulations of NlUG12-encoding mRNA below control levels except for day 7 (down from the JGM influence by 39–53%) (Figure 1). The pattern did not differ across days (all statistics are listed in Table 3). We recorded an interactive effect between DAE and JGM+dsRNA treatments.

**Table 3:** Influence of JGM+dsUGT12 treatment on the number of offspring, hatching rate, and gender ratio.

Treatments	Number of eggs laid	Hatching rate	Gender ratio	PGI (N1/N0)
Control	386.0 ± 25.7b	0.91 ± 0.03a	1.44 ± 0.42ab	96.5
JGM	516.6 ± 56.2a	0.95 ± 0.02a	1.92 ± 0.41a	129.2
JGM+dsGFP	534.8 ± 97.3a	0.94 ± 0.02a	2.02 ± 0.46a	113.7
JGM+dsUGT12	286.4 ± 54.2c	0.83 ± 0.05b	1.65 ± 0.55ab	71.6

*^∗^Means ± SE of five replicates. Means with columns followed by different letters are significantly different at the 5% level (p < 0.05, Tukey test). Control Group, control-♀ × control-♂; JGM Group, JGM-♀ × control-♂; JGM+dsGFP Group, JGM+dsGFP-♀ × control-♂; JGM+dsUGT12 Group, JGM+dsUGT12-♀ × control-♂.*

### dsUGT12 Treatments Alter Protein Contents and Related Parameters in JGM-Treated BPH

**FIGURE 2:**
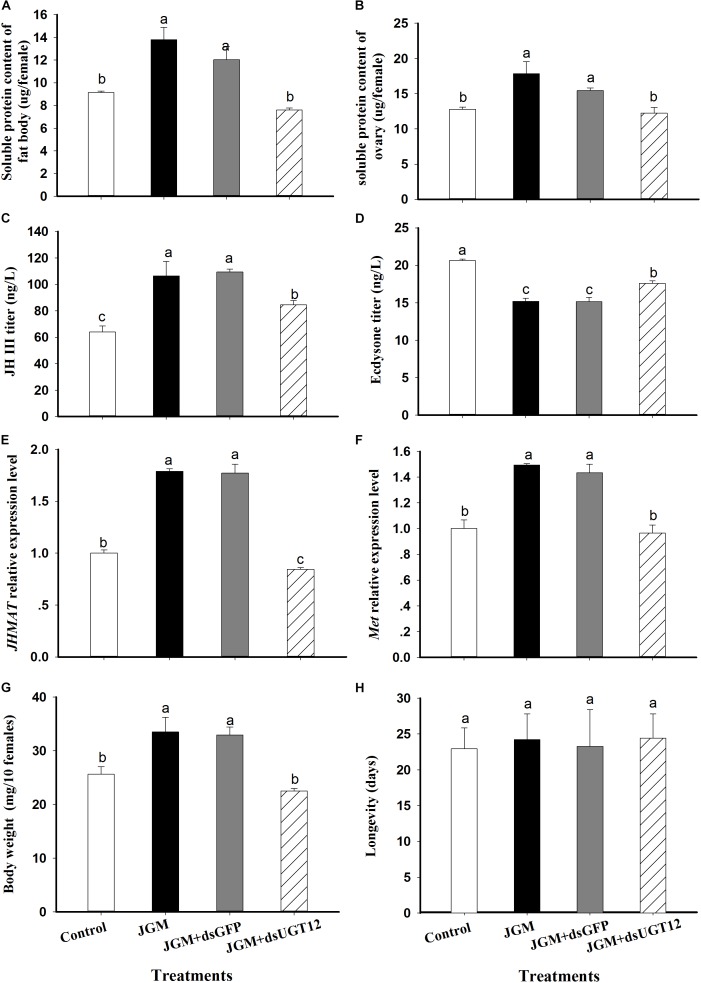
Effects of JGM+dsUGT12 treatments on female physiology at 2 DAE. For all panels, unless noted differently, *n* = 3 independent biological replicates. **(A)** The histogram bars show mean fat body soluble protein content (μg/female,±SE) at 2 DAE. **(B)** The histogram bars show mean ovarian soluble protein content (μg/female). **(C)** the histogram bars show mean JH III titer (ng/L) in adult females (*n* = 3). **(D)** The histogram bars show mean ecdysone titer (ng/L) in adult female. **(E)** The histogram bars show mean *JHAMT* mRNA relative expression level. **(F)** The histogram bars show mean *Met* mRNA relative expression level. **(G)** The histogram bars show mean body weight (mg/10 females). **(H)** The histogram bars show mean longevity of adult females (*n* = 19 independent biological replicates).

Compared to controls, the JGM treatments led to substantially increased fat body and ovarian proteins, JH III titer, accumulations of mRNA encoding JHAMT and Met, and body weights. The treatments led to reduced 20E titers. None of the treatments influenced adult female lifespans (Figure 2). dsUGT12 treatment in nymphs effectively reversed the positive influences of JGM. In general, the dsUGT12 treatment yielded values not different from untreated controls, with the exception of *JHAMT* expression (Figure 2E) with results slightly but significantly lower than control results.

### dsUGT12 Treatments Reduce Egg Deposition in JGM-Treated BPH

**FIGURE 3:**
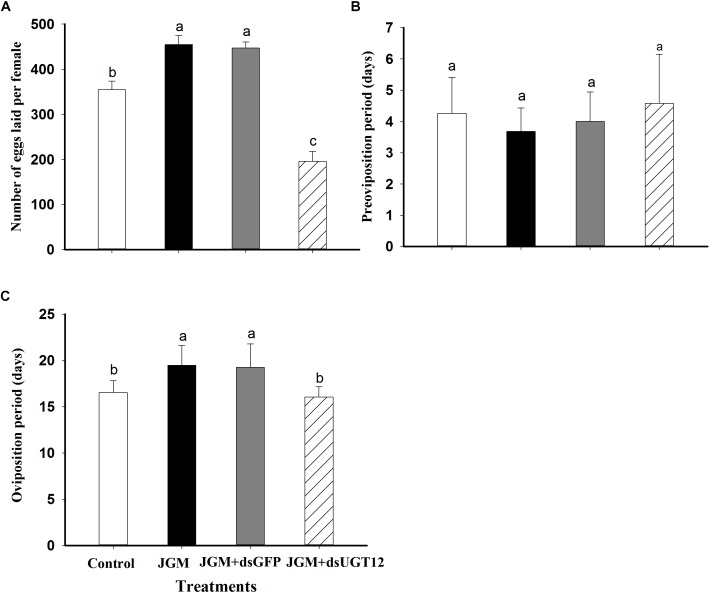
Effects of JGM+dsUGT12 treatment on adult females reproduction parameters. **(A)** The histogram bars show mean numbers of eggs laid (*n* = 19 independent biological replicates). **(B)** The histogram bars show pre-oviposition period (days) (*n* = 19 independent biological replicates). **(C)** The histogram bars show oviposition period (days) (*n* = 19 independent biological replicates).

Untreated control BPH deposited about 370 eggs/female, which was substantially increased in the JGM-treated group. The dsUGT12 treatment led to reduced egg laying in JGM-treated BPHs, significantly below results with untreated controls (Figure 3A). JGM treatments had no influence on the pre-oviposition period (Figure 3B) although the treatments led to significant increase in oviposition period, which was reversed by the dsUGT12 treatment (Figure 3C).

### dsUGT12 Treatments Led to Dysfunctional Ovaries in JGM-Treated BPH

**FIGURE 4:**
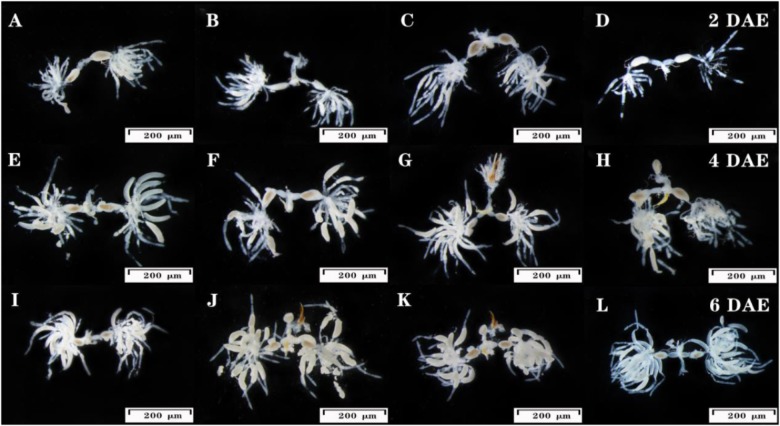
Effects of JGM+dsUGT12 on female reproductive tract at 2, 4, and 6 DAE. The third instar nymphs amenable to JGM spraying were treated with dsUGT12 diet. **(A–L)** reproductive tracts were dissected from mated females and photographed. Ovaries from at least ten females of each group were dissected and observed under a microscope. Scale bar, 200 μm.

dsUGT12 treatment (Figure 4D,H,L) impeded ovarian development compared to JGM-treatments (Figure 4B,C,F,G,J,K) at 2, 4, and 6 DAE. The ovarioles of JGM-treated groups contained two or more banana–shaped oocytes (Figure 4B) at 2 DAE while there were no such oocytes in untreated controls, nor in the JGM+dsUGT12-treated group during the same time period (Figure 4A,D). Ovaries of the untreated control group (Figure 4A,E,I), the JGM-treated group (Figure 4B,F,J), and JGM+dsGFP group (Figure 4C,G,K) contained mature eggs (light brown colored) at 4 and 6 DAE, but the JGM+dsUGT12-treated group contained no mature eggs (Figure 4H,L).

### dsUGT12 Treatments Reversed the Enhancing Influence of JGM on *NlVg* Expression and Vg Synthesis

**FIGURE 5:**
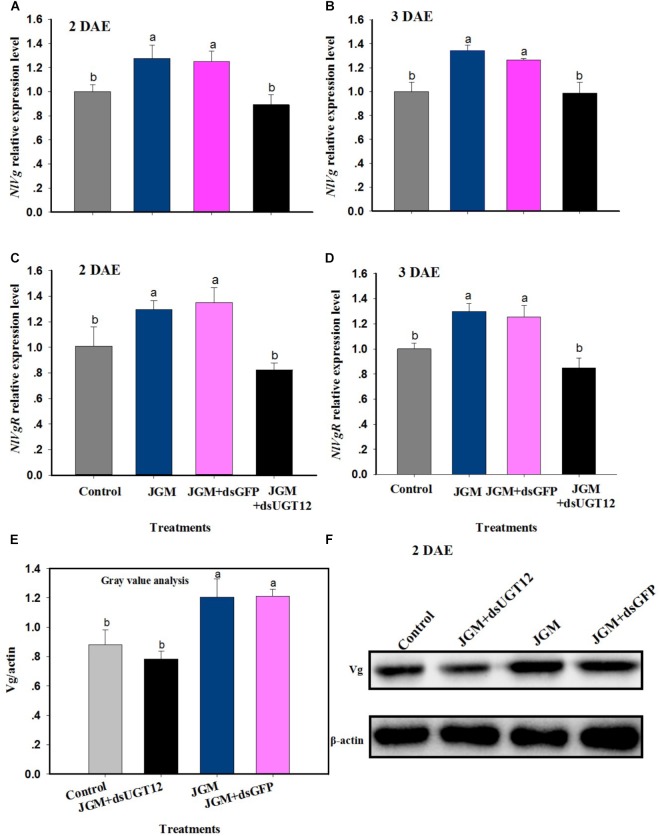
Effects of JGM+dsUGT12 treatment on *NlVg, NlVgR* expression and Vg protein synthesis. **(A**,**B)** The histogram bars show mean *NlVg* expression level at 2 DAE and 3 DAE, respectively. **(C**,**D)** The histogram bars show mean *NlVgR* expression level at 2 DAE and 3 DAE. **(E)** The histogram bars show mean gray values of each band at 2 DAE. **(F)** Effects of JGM+dsUGT12-treated on protein levels of Vg in the fat body of adult females at 2 DAE. Western blot analysis was performed using Vg antibody specific for *N. lugnes*.

Jinggangmycin treatments led to increased accumulations of mRNAs encoding NlVg and NlVgR relative to the controls. The dsUGT12 treatments reversed the enhancing influence of JGM at 2 DAE and 3 DAE (Figure 5A,B). We recorded similar results with *NlVgR* at 2 and 3 DAE (Figure 5C,D). With respect to Vg protein contents, dsUGT12 treatment overcame the JGM-induced results (Figure 5E). Western blot analysis yielded similar results (Figure 5F and Supplementary Figure S1).

### Methoprene Application Rescues *NlUGT12* Expression and Vg Synthesis

**FIGURE 6:**
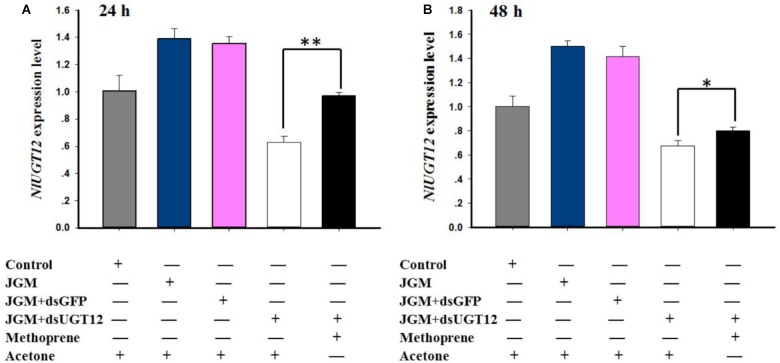
Effects of methoprene topical application on *NlUGT12* expression level. **(A)** The histogram bars show *NlUGT12* expression level at 24 h post administered (^∗∗^ denotes significant difference at *P* < 0.01). **(B)** The histogram bars showed *NlUGT12* expression level at 48 h post administered (The asterisk represents significant difference at *P* < 0.05).

**FIGURE 7:**
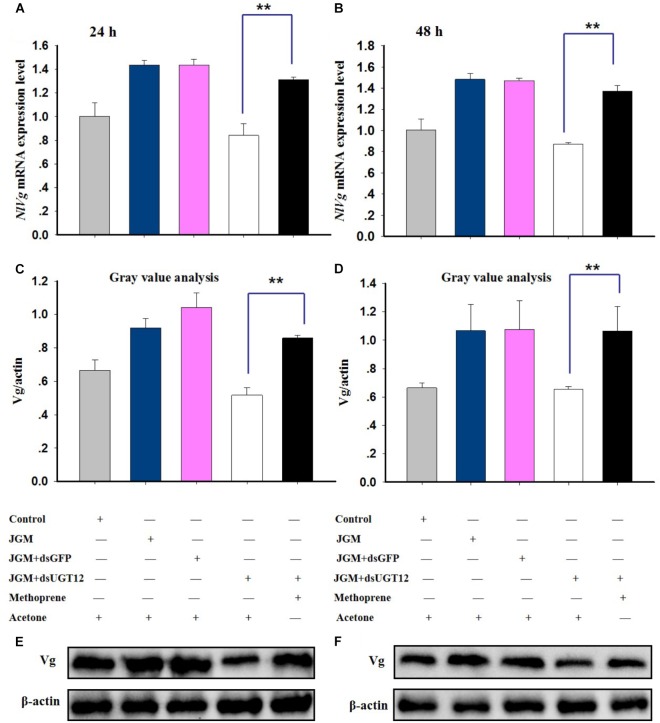
Effects of methoprene topical application on *NlVg* expression and Vg protein expression levels. The histogram bars show *NlVg* expression level at 24 h **(A)** and 48 h **(B)** (^∗∗^ denotes significant difference at *P* < 0.01). The histogram bars show mean gray values of Vg protein levels at 24 h **(C)** and 48 h **(D)** (^∗∗^ denotes significant difference at *P* < 0.01). The Vg protein levels of JGM+dsUGT12-treated female fat bodies was quantified 24 h **(E)** and 48 h **(F)** post methoprene topical application relative to the other groups which received the equivalent amount of acetone.

Jinggangmycin led to increased accumulations of mRNA encoding NlUGT12 which, again, were reversed by the dsUGT12 treatments. Topical methoprene application onto newly emerged adult females partially rescued *NlUGT12* expression in the dsUGT12-treated group at 24 h (Figure 6A) and 48 h post treatment (Figure 6B). Methoprene applications similarly rescued *NlVg* expression (Figure 7A,B) and Vg contents (Figure 7C,D). Western blot analysis yielded similar results (Figure 7E,F and Supplementary Figure S2).

### dsUGT12 Treatment Led to Reduced Egg Laying and Hatch Rates

Compared to untreated controls, JGM treatments significantly increased the number of eggs laid (by 34%), gender ratio (by 33%), and PGI (by 34%). dsUGT12 treatments led to reduced numbers of deposited eggs (by 45%) and egg hatching rates (by 13%), gender ratio (by 14%), and PGI (by 45%) in the JGM+dsUGT12 treated group compare to JGM treated group (Table 2). dsGFP treatments had no effect on JGM-enhanced egg deposition (Table 2). None of the treatments influenced the BPH gender ratios.

## Discussion

The results presented in this paper strongly support our hypothesis that *NlUGT12* is a positive modulator of BPH reproductive biology. Several points are germane to the point. First, JGM treatments led to increased fat body and ovarian protein contents, increased JHIII titers, decreased 20E titers, increased JHAMT and Met expression, increased body weights, increased egg deposition and duration of the oviposition period, elevated expression of *Nlvg* and *NlvgR* and production of Vg protein, and elevated *UGT12* expression. Second, a dsRNA construct specific to NlUGT12 effectively reversed the JGM enhanced influence. Third, JH analog methoprene treatment partially rescued dsUGT12 effect. We infer that *NlUGT12* acts via its cognate protein NlUGT12, as a positive modulator of BPH reproduction.

Overall, our results tend to show that NlUGT12 modulates fecundity and population growth in *N. lugens*, probably by regulating JH and Vg synthesis. Indeed, JGM is a very broadly-applied treatment for rice sheath blight (*R. solani*) control in China (Peng et al., 2014). These treatments lead to increased egg deposition. Because egg development is mediated by JH, we considered the possibility that the JGM effect on reproduction acts through JH. JGM exposure leads to increased JH, but not 20E, titers and to increases in mRNA encoding two JH-related proteins, JHAMT and the JH receptor, Met. JHAMT acts at the final step in converting inactive precursors into active JH and increases in JHAMT expression is consistent with increased JH titers. Similarly, increases in Met expression are consistent with enhancing the physiological functioning of JH. 20E titers were decreased under JGM exposure, from which we infer the JGM effects are focused on reproductive, rather than developmental events. Most results indicate that suppressing *NlUGT12* expression reverses the enhancing influences of JGM. We considered the possibility of a direct connection between *NlUGT12* and JH actions, from which we determined the reversing influence of a JH analog, methoprene, on dsRNA-mediated *NlUGT12* suppression.

Insect eggs reflect heavy resource investments, particularly proteins. Increased amounts of fat body protein are consistent with increased Vg synthesis, transport, and receptor-mediated ovarian uptake, a necessary foundation of increased egg laying. The increased protein supports increased egg production and deposition, which is seen, also, in higher numbers of deposited eggs. These JGM-mediated increases may be seen in increased body weights, particularly in females. We determined the influence of JGM on oviposition periods because insect egg-laying processes take time. Although it varies according to the rice developmental stage, BPHs typically lay eggs in groups, in rows usually in rice sheaths but also on leaves. We recorded increased oviposition periods, which is consistent with increased egg deposition. We considered egg hatch rates as an indicator of egg quality, which was not influenced by JGM.

UDP-glucuronosyltransferases act in multiple processes, including detoxification of ingested plant allelochemicals and inactivation of steroid hormones (Ahmad and Hopkins, 1993a; Kojima et al., 2010; Ahn et al., 2011). Phylogenetic analysis (Hughes, 2013) indicates the closest relatives of baculovirus EGT are the UGT33 and UGT34 families of lepidopteran UGTs. Baculoviral EGT disrupts the hormonal balance of insect hosts by catalyzing the inactivating conjugation of 20Es with a sugar moiety from UDP-glucose or UDP-galactose (Evans and O’Reilly, 1999), which leads to abnormal larval growth (Shikata et al., 1998; Hughes, 2013). Because UPDs act in 20E metabolism, we infer that *NlUGT12* positively modulates reproduction and the JGM-driven increases in reproduction via influencing developmental hormone titers.

The idea that NlUGT12 acts via influencing hormone titers is strongly supported by our findings. Silencing *NlUGT12* led to diminished JH III titers and increased 20E titers, relative to the outcomes of JGM and JGM+dsGFP treatments, from which we infer that NlUGT12 directly influences developmental hormone titers. mRNAs encoding JHAMT, which acts in JH biosynthesis and Met, the receptor responsible for JH actions, were diminished in BPHs following dsNlUGT12 treatments, again, supporting our view of *NlUGT12* as a central player in BPH hormonal homeostasis. The *Vg* and *VgR* directly act in insect fecundity. Their synthesis, transport and uptake are regulated mainly by JHs and, again, dsUGT12 treatments in JGM treated group led to reduced JH titers, which reduces *Vg* and *VgR* expression and subsequent ovarian Vg uptake. We infer that NlUGT12 exerts its actions via developmental hormones.

Topically applied methoprene, a JH analog, reversed the influences of the JGM+dsUGT12 treatments on *NlUGT12* and *NlVg* expression, and on Vg synthesis. Our interpretation is that *NlUGT12* influences JH synthesis, which regulates *NlVg* expression and Vg protein synthesis. The reduced protein contents of fat bodies and ovaries in JGM+dsUGT12-treated females may preclude synthesis of yolk protein from vitellogenin, accompanied by restricted ovarian development and reproduction.

## Author Contributions

LG designed the research. SZ, YZ, ZZ, and HG conducted the experiments. LG wrote the first draft of the manuscript. QS and DS revised the final draft of the manuscript.

## Conflict of Interest Statement

The authors declare that the research was conducted in the absence of any commercial or financial relationships that could be construed as a potential conflict of interest.

## Footnotes

1 https://imagej.nih.gov/ij/
