# Supplementary material for: Jinggangmycin-Induced UDP-Glycosyltransferase 1-2-Like Is a Positive Modulator of Fecundity and Population Growth in Nilaparvata lugens (Stål) (Hemiptera: Delphacidae)
Source: Front Physiol. 2019 Jun 21;10:747. doi: 10.3389/fphys.2019.00747 (PMC6598453; doi:10.3389/fphys.2019.00747)
Supplement: Supplementary file 1 [file Table_1.docx]

FigureS1:


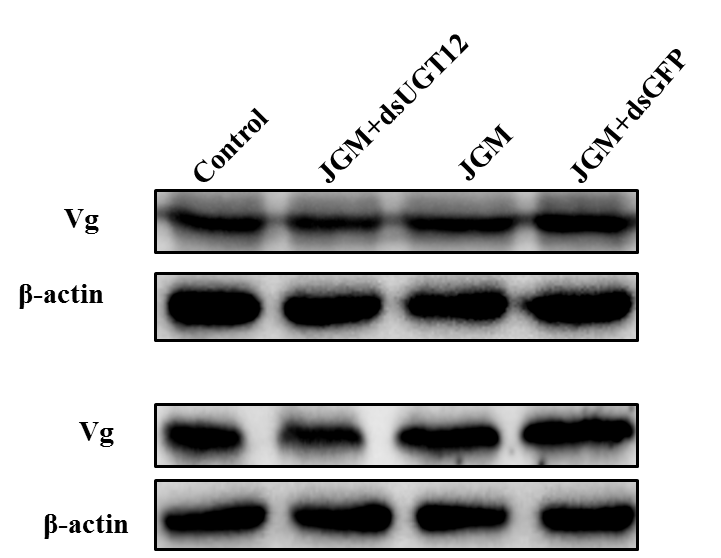


**2 DAE**

**B**

**A**

Figure.S1 Effects of JGM+dsUGT12 treatment on Vg protein synthesis. Panel (B-C) showed other two replicated of western blot in Figure.5F at 2 DAE.

Figs2:


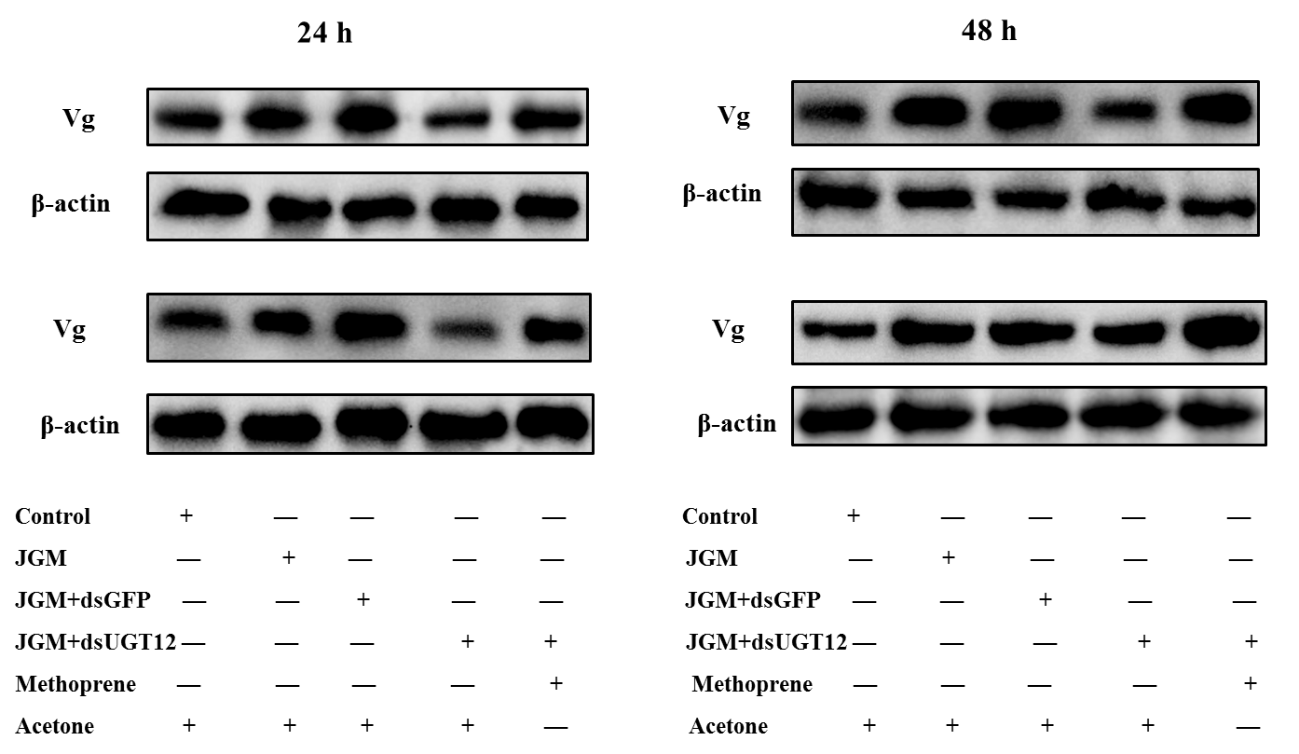


**D**

**C**

**B**

**A**

Figure.S2 Effects of methoprene topical application on Vg protein synthesis. Panel(A-B) showed other two replicated of western blot in Figure. 7E at 24 h. Panle (C-D) showed other two replicated of western blot in Figure. 7F at 48 h.
